# The impact of light/dark regimes on structure and physiology of *Chlorella vulgaris* biofilms

**DOI:** 10.3389/fmicb.2023.1250866

**Published:** 2023-10-24

**Authors:** Yan Gao, Olivier Bernard, Andrea Fanesi, Patrick Perré, Filipa Lopes

**Affiliations:** ^1^Laboratoire Génie des Procédés et Matériaux (LGPM), CentraleSupélec, Université Paris-Saclay, Gif-sur-Yvette, France; ^2^Biocore, Inria Sophia Antipolis Méditerranée, Université Nice Côte d'Azur, Valbonne, France; ^3^Laboratoire Génie des Procédés et Matériaux (LGPM), Centre Européen de Biotechnologie et de Bioéconomie (CEBB), CentraleSupélec, Université Paris-Saclay, Pomacle, France

**Keywords:** microalgae, biofilm, light cycle, photoinhibition, architecture

## Abstract

**Introduction:**

Biofilm-based microalgae production technologies offer enormous potential for improving sustainability and productivity. However, the light pattern induced by these technologies is a key concern for optimization.

**Methods:**

In this work, the effects of light/dark cycles on architecture, growth, and physiology of *Chlorella vulgaris* biofilms were assessed in a millifluidic flow-cell with different time cycles (15 s to 3 min) keeping the average light constant at 100 μmol·m^−2^·s^−1^.

**Results and discussion:**

Results showed that photoinhibition can be mitigated by applying a light fraction of 1/3 and a cycle time of 15 *s*. By contrast, when the cycle time is extended to 90 s and 3 min, photoinhibition is high and photoefficiency dramatically decreases. To cope with light stress, cells acclimate and organize themselves differently in space. A high peak light (500 μmol·m^−2^·s^−1^) triggers a stress, reducing cell division and inducing clusters in the biofilm. This work provides guidelines for optimizing rotating microalgae production systems in biofilms and assesses the minimum rotating frequency required to maintain the net growth rate close to that of continuous light of the same average intensity, mitigating photo-inhibition. The overall gain in productivity is then provided by the total surface of the biofilm turning in the illuminated surface area.

## 1. Introduction

Microalgae are a promising source of food, feed, pigments, antioxidants, and at longer term molecules for green chemistry and biofuel (Mata et al., 2010). They are mainly cultivated in suspension-based systems, such as open ponds or photobioreactors (Borowitzka, 1999) with the drawbacks of a high energy and environmental cost for culture mixing, harvesting, and dewatering (Milledge and Heaven, 2013). Some species of microalgae can also grow attached to a support, in complex structures called biofilms (De Beer and Stoodley, 2006). A biofilm is an assemblage of microbial cells irreversibly associated with a surface and enclosed in a matrix of extracellular polymers substances (EPS) (De Beer and Stoodley, 2006). Its three-dimensional structure is strongly heterogeneous in terms of physical, chemical and metabolic properties (De Beer and Stoodley, 2006).

Biofilm-based technologies are an emerging process for microalgae cultivation which has been shown to reach higher productivity (Gross and Wen, 2014; Wang et al., 2017) with lower land and water demands compared with conventional systems (Ozkan et al., 2012; Liu et al., 2013). Harvesting also becomes much simpler by scraping the biofilm (Gross et al., 2015a).

Biofilm structure, growth and cell physiology are strongly affected by environmental and operating factors (Fanesi et al., 2021, 2022). Among them, light is a major parameter to be considered when designing and operating biofilm-based systems. Photosynthetic Photon Flux Density (PPFD) from 100 to 400 μmol·m^−2^·s^−1^ is generally reported as the optimal range to maximize the biofilm growth rate (Liu et al., 2013; Mantzorou and Ververidis, 2019). Higher PPFD [e.g., over 2,000 μmol·m^−2^·s^−1^ at noon in summer (Liu et al., 2013)] induce photoinhibition and ultimately cell death. One of the technical solutions to avoid photodamage consists in diluting light in time by exposing microalgae cells to alternating light/dark (L/D) cycles (Liao et al., 2014; Abu-Ghosh et al., 2016; Toninelli et al., 2016). This strategy can be implemented by developing biofilms in partially submerged revolving reactors where sessile cells are periodically submitted to light and darkness (Bernstein et al., 2014; Gross et al., 2015b).

Fluctuating light regimes are characterized by several parameters:

- the cycle time (*T*) which is the total period of a light/dark (L/D) cycle,
- the maximum (or peak) PPFD (*I*_*peak*_) during one cycle,
- the time-averaged PPFD (*I*_*ave*_) corresponds to the light dose received by a cell during a cycle time, and
- the light fraction (ε) or duty cycle which is the fraction of time that cells are exposed to light in one cycle.

These parameters affect photosynthetic efficiency, growth, biomass and products synthesis. Photosynthetic growth in fluctuating light is generally considered to be bounded by two extreme regimes. At high light frequency (>100 Hz, short cycle time) growth rate is governed by the time-averaged PPFD [*full-light-integration* (Vejrazka et al., 2011)]. At low frequency (<0.01 Hz, long cycle time) growth takes place during the light periods at a rate similar to that at continuous light [*no-light-integration* (Xue et al., 2011; Graham et al., 2017)]. *Full-light-integration* was first reported by Emerson and Arnold (1932) using cycle times on the scale of milliseconds in suspended algal cultures (Sforza et al., 2012; Schulze et al., 2020). With such short periods, photosystems can use the high PPFD with minimal inhibition. *Full-light-integration*, corresponding to the highest photon use efficiency, is achieved when applying fast flashing light (μs–ms) even when the cells are exposed to inhibiting peak PPFD (greater than the optimal light intensity) (Xue et al., 2011; Schulze et al., 2020). By contrast, for cycle times longer than hundreds of seconds, photoinhibition is high, and photoefficiency decreases dramatically (Xue et al., 2011; Graham et al., 2017). These phenomena have been widely studied for planktonic cultures, but only few studies have focused on biofilm growth with L/D cycles (Toninelli et al., 2016; Martín-Girela et al., 2017; Grenier et al., 2019).

More investigations are required to better understand the mechanisms (photoacclimation, photoregulation) involved in biofilm development and optimize L/D cycles to enhance growth and ultimately productivity (Toninelli et al., 2016; Zhang et al., 2019). Beyond growth rate and productivity, physiological parameters such as cell size, pigment content and photosynthetic activity must be considered. In the photoacclimation process, extensively described for planktonic cultivation, cellular chlorophyll decreases when cells are submitted to a high PPFD (MacIntyre et al., 2002). Under fluctuating light regimes, chlorophyll content seems mainly dependent on the average light intensity, rather than on peak light (Combe et al., 2015). The impact of intermittent light on chlorophyll content of biofilm cultures has been much less reported (Zhang et al., 2019). Cell volume is also affected by light intensity in suspended cultures, with larger size with increased PPFD (Winokur, 1948; Claustre and Gostan, 1987). Cell size for fluctuating light has been though less studied. Combe et al. (2015), with *Dunaliella salina* did not record any cell size change in fluctuating regime, while Vejrazka et al. (2011) with *Chlamydomonas reinhardti* observed that cell size increased at faster light variations, eventually tending to similar features as in continuous light. The maximum quantum yield (*F*_*v*_/*F*_*m*_) provides information regarding the PSII physiological state, indicating if there is a stress on PSII (Masoj́ıdek et al., 2013). The *F*_*v*_/*F*_*m*_ of healthy microalgae cultures (planktonic cultures) usually ranges from 0.7–0.8 depending on the microalgae species, while lower values suggest stress (Masoj́ıdek et al., 2013). Flashing light frequency and peak PPFD have been shown to impact strongly *F*_*v*_/*F*_*m*_ in suspended cultures (Sforza et al., 2012).

The effects of L/D cycles on biofilm cultures (growth and physiology) have been poorly studied. The goal of this work is therefore to better understand the effect of L/D cycles on biofilm growth (cell number and size), structure dynamics and sessile cell physiology. Physiological and photosynthetic parameters including *F*_*v*_/*F*_*m*_, α, *rETR*_*max*_, chlorophyll-a content and average cell volume were monitored over time. In one of our previous works, growth was not enhanced for light cycles of 30–40 min (Grenier et al., 2019). Here, shorter cycle times ranging from 15 s to 3 min, with light fraction of 1/3 and 1/5 were tested with an average PPFD of 100 μmol·m^−2^·s^−1^. *Chlorella vulgaris* biofilms exposed to continuous light show a decrease in photosynthetic rate at intense light of 500 μmol·m^−2^·s^−1^, revealing a strong photoinhibition (GAO et al., 2023). In this study, we also intend to verify if photoinhibition can be mitigated with intermittent illumination conditions, with a simplified single species biofilm model.

Here we demonstrate that sessile microalgae do respond to L/D cycles through physiological adjustments (size, pigment content, photosynthetic activity) to optimize photosynthetic performance and protect themselves from excess of energy. General mechanisms already described for suspended cells are involved (photoacclimation, photoregulation, clustering) but, a unique photoprotection mechanism involving cell organization in photosynthetic sessile cells is suggested here. Our experimental work eventually provided clues to choose the most appropriate operating conditions (cycle time, duty cycle) to run a rotating biofilm system.

## 2. Materials and methods

### 2.1. Microalgae species and inoculum culture

*Chlorella vulgaris* SAG 211-11B (Göttingen, Germany) was cultivated in 3N-Bristol medium (Bischoff, 1963) in a 100 mL glass tube with a working volume of 70 mL in a PSI MC1000 multicultivator (Photon systems instruments, Drásov, Czech Republic) at 25°*C*. CO_2_ supply and mixing were carried out by constant air bubbling. The suspended cultures were maintained in the exponential phase (2 × 10^6^−3 × 10^6^ cells·mL^−1^) and photoacclimated for 2 weeks to the respective light regimes (see Section 2.3) that were further employed in the biofilm experiments.

### 2.2. Biofilm system set-up

*C. vulgaris* biofilms were cultivated in a flow-cell system similar to that used by Le Norcy et al. (2019). Biofilms were grown in 2-channel Poly-methyl methacrylate (PMMA) flow-cell with dimensions of 40 × 6 × 3 mm (length × width × height) where the substratum was represented by a glass coverslip (see Supplementary material). Before inoculation, the system was first sterilized by sodium hypochlorite solution (0.5%) for 3 h and then flushed with 2 L autoclaved distilled water. It was finally filled with 3N-Bristol medium overnight. For inoculation, 3 mL pre-diluted inoculum culture with a cell concentration of 7 × 10^5^cells·mL^−1^ was injected into each channel through an in-line luer injection port (Ibidi GmbH, Germany). After 24 h without medium flow to ensure cell attachment, fresh medium was added to the flow-cell system at 0.1 mL·min^−1^ (velocity = 0.093 mm·s^−1^; Reynolds number = 0.37 and shear stress = 2.3 mPa). The temperature was controlled at 24 ± 1°*C*. pH in the outlet flow, measured at days 2, 7, and 15, was similar to that of the inlet medium (6.5). Carbon limitation is therefore not expected.

### 2.3. Light regimes

Light (emitting diodes, MEAN WELL ENTERPRISES CO., LTD. ELG-240-24, China) was supplied either continuously at 100 μmol·m^−2^·s^−1^ or through L/D cycles (average PPFD of 100 μmol·m^−2^·s^−1^) and measured with a Quantitherm PAR/Temp Sensor (Hansatech Instruments Ltd., Norfolk, UK) within the detected spectra range from 400 nm to 700 nm. A first set of experiments were run with different L/D cycles (L/D of 5 s/10 s, 30 s/60 s, 1 min/2 min) with peak PPFD of 300 μmol·m^−2^·s^−1^ (for instance, “300-5s-0-10s” refers to 300 μmol·m^−2^·s^−1^ for 5 s and darkness for 10 s. “100 cont” refers to continuous light at 100 μmol·m^−2^·s^−1^). A second set of assays were then carried out with peak PPFD of 500 μmol·m^−2^·s^−1^ and L/D of 5 s/20 s (see Table 1). The number of independent assays were 5, 3, 7, 4, 3 for 100 cont, 300-5s-0-10s, 300-30s-0-60s, 300-1min-0-2min, and 500-5s-0-20s, respectively. For each independent assay, six replicates were performed (meaning 3 parallel flow-cells with a total number of 6 channels)

**Table 1:** Illumination profiles applied to the flow-cell biofilm system.

**Light regimes notation**	**Peak PPFD (μmol·m^−2^·s^−1^)**	**Average PPFD (μmol·m^−2^·s^−1^)**	**Light fraction ε**	***T*_*L*_**	***T*_*D*_**	***T***
100 cont	106 ± 2	106	1	/	/	/
300-5s-0-10s	310 ± 5	103	1/3	5 s	10 s	15 s
300-30s-0-60s	310 ± 5	103	1/3	30 s	60 s	90 s
300-1min-0-2min	310 ± 5	103	1/3	1 min	2 min	3 min
500-5s-0-20s	496 ± 3	99	1/5	5 s	20 s	25 s

*T*_*L*_ represents light phase duration, while *T*_*D*_ is the dark phase duration.

### 2.4. Biofilm structure

Biofilm development under different light conditions was monitored *in situ* and non-destructively using an inverted Zeiss LSM 700 Confocal Laser Scanning Microscope (CLSM, Carl Zeiss microscopy GmbH, Jena, Germany). All biofilm 3D structures were acquired through a LD Plan-Neofluar 20 × 0.4 Korr M27 objective with a 0.4 N.A. (numerical aperture). Each slice has a frame size of 512 × 512 pixels and image size of 638.9 × 638.9 μm^2^. Pixel size is 1.25 μm. Each z-step is 3.94 μm. One laser channel was applied to detect microalgal chlorophyll-a autofluorescence which was excited by 5-mW solid-state diode laser at 639 nm and detected at 615 nm after the long pass (CP) filter. The laser power was set at 1.0 and the gain (master) of the channel was set at 650.

Biofilm of each flow-cell channel was measured *in situ* at five positions along the channel to obtain an average index of the biofilm structure. Measurements were carried out every 24 h to follow biofilm structural dynamics. A set of structural parameters were obtained afterwards by image analysis {ImageJ 1.48v software (Schneider et al., 2012) and the plug-in COMSTAT 2.1 [Technical University of Denmark (Heydorn et al., 2000)]: biovolume (μm^3^·μm^−2^), maximum thickness (μm), average thickness (μm), roughness (A.U.)}. It is worth noting that autofluorescence of cells is related to chlorophyll within chloroplast. However, to be in accordance with the terminology presented in most of the literature (Fanesi et al., 2019), we consider the increase of autofluorescence as cells proliferation, although autofluorescence does not quantify the exact cell numbers.

### 2.5. Biomass

Cell density measurement was carried out using a destructive method. Biofilm cells were taken out of each flow-cell channel on day 2, 7, and 15, respectively, by flushing Bristol medium through it, at least twice. Cell concentration was afterwards measured using flow cytometry [Guava easyCyte 5 flow cytometer (Millipore corporation 25801 Industrial Blvd Hayward, CA94545)] with chlorophyll-a excitation at 488 nm and fluorescence detection at 680 nm. Sample's cell concentration was kept in the range of 1 × 10^4^ to 6 × 10^5^ cells·mL^−1^ by medium dilution for measurements. The areal cell density was obtained from total cell number divided by the substratum surface of the channel (0.24 cm^2^).

### 2.6. Light transmittance

Light transmission through the biofilm was calculated daily based on the difference between light intensity above and below the flow-cell (Equation 1) measured by the Quantitherm PAR/Temp Sensor.


(1) $$\begin{array}{l} Light\;attenuation=\frac{I_{in}-I_{out}}{I_{in}}\times 100\% \end{array}$$

where *I*_*in*_ refers to incident light on the top of the flow-cell, *I*_*out*_ refers to output light through the channel with biofilm (mean of three positions' outputs along the channel).

### 2.7. The specific growth rate

Biofilm specific growth rate was determined using light transmittance data. The light transmittance in biofilms follows the Lambert-Beer Law:


$$\begin{array}{l} I_{out}=I_{in}e^{-k\cdot X}, \end{array}$$

where X is the biomass (g·m^−2^), *k* is the light extinction coefficient (m^2^·*g*^−1^). Thus:


$$\begin{array}{l} X=\frac{1}{k}ln\frac{I_{in}}{I_{out}}. \end{array}$$

Accordingly, the specific growth rate (μ, *d*^−1^) based on light transmittance is the maximum slope of the regression between $ln\left(ln\frac{I_{in}}{I_{out}}\right)$ and time *t* (at least four data points were used). The specific growth rate μ stands for the average net growth rate during a cycle, resulting from the balance between gross photosynthesis in light phase and respiration (*R*, *d*^−1^) in light and darkness. The gross growth rate in light phases (μ_*L*_, *d*^−1^) comes from gross photosynthesis. Therefore,


(2) $$\begin{array}{l} \mu_{L}=\frac{\mu +R}{\varepsilon }. \end{array}$$

### 2.8. Physiological parameters

Sessile cell physiology (cell volume, chlorophyll content) and *F*_*v*_/*F*_*m*_ were assessed by off-line measurements on day 2, day 7, and day 15, respectively. Biofilm cells were extracted from each channel as previously described. Physiology of the inoculum culture (day 0) was analyzed to compare with that of biofilm cells.

#### 2.8.1. Cell volume

Cell volume was measured by image acquisition through microscope imaging (Brightfield in transmission mode) and subsequent image analysis (software ImageJ v1.48). On day 2, cell observation and volume estimations *in situ* were possible due to the low cell density. On days 7 and 15, the cells were withdrawn, concentrated (to 1 × 10^8^−2 × 10^8^ cells·mL^−1^ by centrifugation at 14,500 rpm) and observed by optical microscopy. 2D images were first obtained by the inverted Zeiss LSM 700 Confocal Laser Scanning Microscope (CLSM, Carl Zeiss microscopy GmbH, Jena, Germany) with Zen 10.0 software black edition (Carl Zeiss microscopy GmbH, Jena, Germany). LD Plan-Neofluar 20 × 0.4 Korr M27 objective with a 0.4 N.A. was used to take the picture with a frame size of 256 × 256 pixels (pixel size: 0.32 μm) and image size of 82.2 × 82.2 μm^2^. On the other hand, optical track channel (TV1) was used for optical microscopy acquisition. The 2D image including cells were analyzed by ImageJ v1.48 software directly. The image type was chosen as 8-bit, the threshold adjusted. After making binary of the image and all cells being filled in black with a white background, the area of each cell was analyzed by carrying out “Analyze particles.” The cell size limit was set as 0- infinity with the pixel units concerned. Assuming all cells to be spheres of similar diameter, the cell volume (μm^3^) can be determined from the cell area (Equation 3):


(3) $$\begin{array}{l} Cell\;volume=\frac{4}{3}\cdot A\cdot \sqrt{\frac{A}{\pi }} \end{array}$$

where *A* (μm^2^) is the area of the microalgae cell, which is determined from image analysis.

#### 2.8.2. Chlorophyll-a content

Chlorophyll-a was extracted from microalgal cells using Dimethyl-sulphoxide (DMSO) (Li et al., 2021). First, cells (range: 4 × 10^6^−10 × 10^6^ cells) were filtrated on glass fiber filters (Fisher Scientific, size: 47 mm, EU). The filter was cut into 5 mm strip and then submerged in 1 mL DMSO in 1.5 mL Eppendorf tube and mixed for 5 min. Chlorophyll-a extraction was carried out for 40 min at room temperature in the dark. After being centrifuged for 5 min with 1300 rpm, the supernatant was transferred to a 1.5 mL cuvette for absorbance measurement by a UV Visible Spectrophotometer (Thermo Fisher Scientific, EVOLUTION 60s, China). Chlorophyll-a (μg·mL^−1^) was calculated according to Equation 4 (Li et al., 2021):


(4) $$\begin{array}{l} Chlorophyll-a=12.19\cdot abs665-3.45\cdot abs649 \end{array}$$

Where abs665 and abs649 refer to the absorbance at wavelength of 665 nm and 649 nm, respectively. Chlorophyll-a content per cell (pg·cell^−1^) and per cell volume (fg·μm^−3^) were subsequently calculated.

#### 2.8.3. The photosynthetic parameters

The photosynthetic efficiency of biofilms was measured by using a portable pulse amplitute modulation (PAM) fluorometer (AquaPen, Photon Systems Instruments, AP110-C, Czech Republic) as described by Li et al. (2021). Before each determination, according to the chlorophyll-a content, the concentration of harvested biofilm sample was adjusted to the range of 5 × 10^5^–1 × 10^6^ cells·mL^−1^ in 3 mL working volume in a 4 mL cuvette. After 15 min of dark adaptation, the samples were exposed to a stepwise increase of seven actinic lights (from 0 to 1,000 μmol·m^−2^·s^−1^) applied every 60 s. The wavelength for fluorescence excitation was 455 nm while for fluorescence detection was 667 nm–750 nm. The maximum quantum yield (*F*_*v*_/*F*_*m*_) and the effective quantum yield ($\Delta F/F_{m}^{\prime }$) were calculated by:


(5) $$\begin{array}{l} F_{v}/F_{m}=\left(F_{m}-F_{0}\right)/F_{m}, \end{array}$$

(6) $$\begin{array}{l} \Delta F/F_{m}^{\prime }=\left(F_{m}^{\prime }-F\right)/F_{m}^{\prime }, \end{array}$$

where *F*_0_ is the minimum fluorescence yield determined after dark adaptation; *F*_*m*_ is the maximal fluorescence measured after excitation by a 0.8 s saturation light pulse with PPFD of 3,000 μmol·m^−2^·s^−1^. *F*_*v*_ is the difference between *F*_*m*_ and *F*_0_. *F* and $F_{m}^{\prime }$ are the minimum and maximum fluorescence during illumination.

The relative electron transport rate (*rETR*) was calculated by:


(7) $$\begin{array}{l} rETR=\Delta F/F_{m}^{\prime }\times PAR\times 0.5, \end{array}$$

where PAR is the photosynthetic active radiation at each step and 0.5 is a factor assuming that two photons are required for linear electron transfer. Light curves were quantitatively compared using the parameters of the maximum rate of relative ETR (*rETR*_*max*_), the initial slope of the curves (termed α, representing the maximum light utilization coefficient), and the saturation PAR (*E*_*k*_, *E*_*k*_ = *rETR*_*max*_/α) obtained by fitting the *rETR* versus PAR curves with the rectangular hyperbola [$rETR=rETR_{max}\times \left(1-e^{-\alpha \times PAR/rETR_{max}}\right)$] (Ralph and Gademann, 2005).

### 2.9. Haldane curve fitting

The Haldane model applied in Bernard and Rémond (2012) was used to represent the influence of light on phytoplankton gross growth rate, as Equation 8.


(8) $$\begin{array}{l} \mu_{L}\left(I\right)=\mu_{max}\frac{I}{I+\frac{\mu_{max}}{\alpha }{\left(\frac{I}{I_{opt}}-1\right)}^{2}} \end{array}$$

where μ_*max*_ is the maximal gross growth rate at optimal light intensity (*I*_*opt*_, μmol·m^−2^·s^−1^). α (m^2^·μmol^−1^) is the initial slope of the curve.

Here, we counted respiration loss (R, *d*^−1^) in the net growth rate under intermittent light regimes as Equation 9.


(9) $$\begin{array}{l} \mu \left(I\right)=\mu_{L}\left(I\right)\cdot \varepsilon -R \end{array}$$

The respiration rate is assumed to be constant with the value provided in Grenier et al. (2019), whatever the light condition (see Supplementary material for further details). The Matlab Nelder-Mead simplex direct search minimization algorithm (*fminsearch*) was used to fit the data curve and identify the parameters. The students test was used to compare predictions and experimental results at a significance level of *p* < 0.05.

Growth yield (d^−1^·μmol^−1^·m^2^·s) with cycle time-averaged light input is calculated by Equation 10:


(10) $$\begin{array}{l} Yield=\frac{\mu \left(I\right)}{I\cdot \varepsilon } \end{array}$$

Net footprint productivity (*P*_*f*_) in revolving systems with various designs (different ε) can also be calculated without photoinhibition mitigation.


(11) $$\begin{array}{l} P_{f}=\mu \left(I\right)\cdot \frac{S_{T}}{S_{f}}\cdot \rho \cdot h. \end{array}$$

where *S*_*T*_ (m^2^) is the total area available for biofilm growth; *S*_*f*_ (m^2^) is the footprint of the biofilm system (note that $\frac{S_{T}}{S_{f}}=\frac{1}{\varepsilon }$); ρ (g·m^−3^) is the dry weight-based volumeric biomass density [1.40 ·10^5^ g·m^−3^ from Grenier et al. (2019)]; h (m) is the highest maximum thickness of biofilms observed in this research. More parameter values can be found in the Supplementary material.

### 2.10. Statistics

Results were presented as mean and standard deviation. One-way or two-way ANOVA was performed by GraphPad prism 8.0 to test the statistically significant difference of means between different light regimes and time points. The level of significance was set at 0.05.

## 3. Results and discussion

### 3.1. Alternating light regimes affects biofilm structure

**Figure 1:**
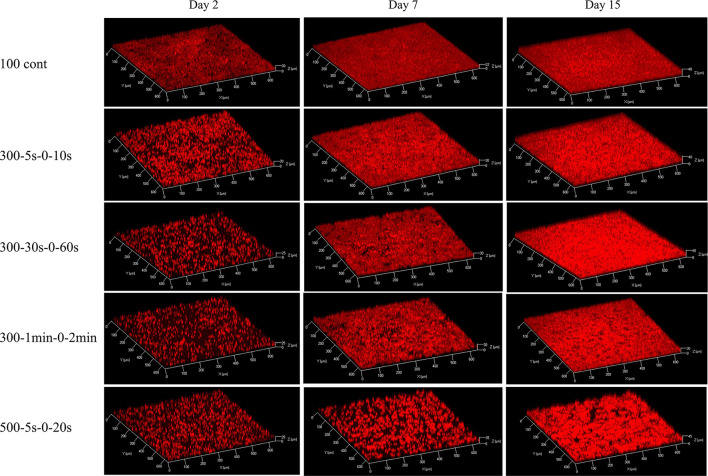
3D structures of biofilms at day 2, day 7, and day 15, under five light regimes: 100 continuous, 300-5s-0-10s, 300-30s-0-60s, 300-1min-0-2min, 500-5s-0-20s, respectively. The red signal corresponds to the auto-fluorescence of chlorophyll which is used as biomass indicator.

**Figure 2:**
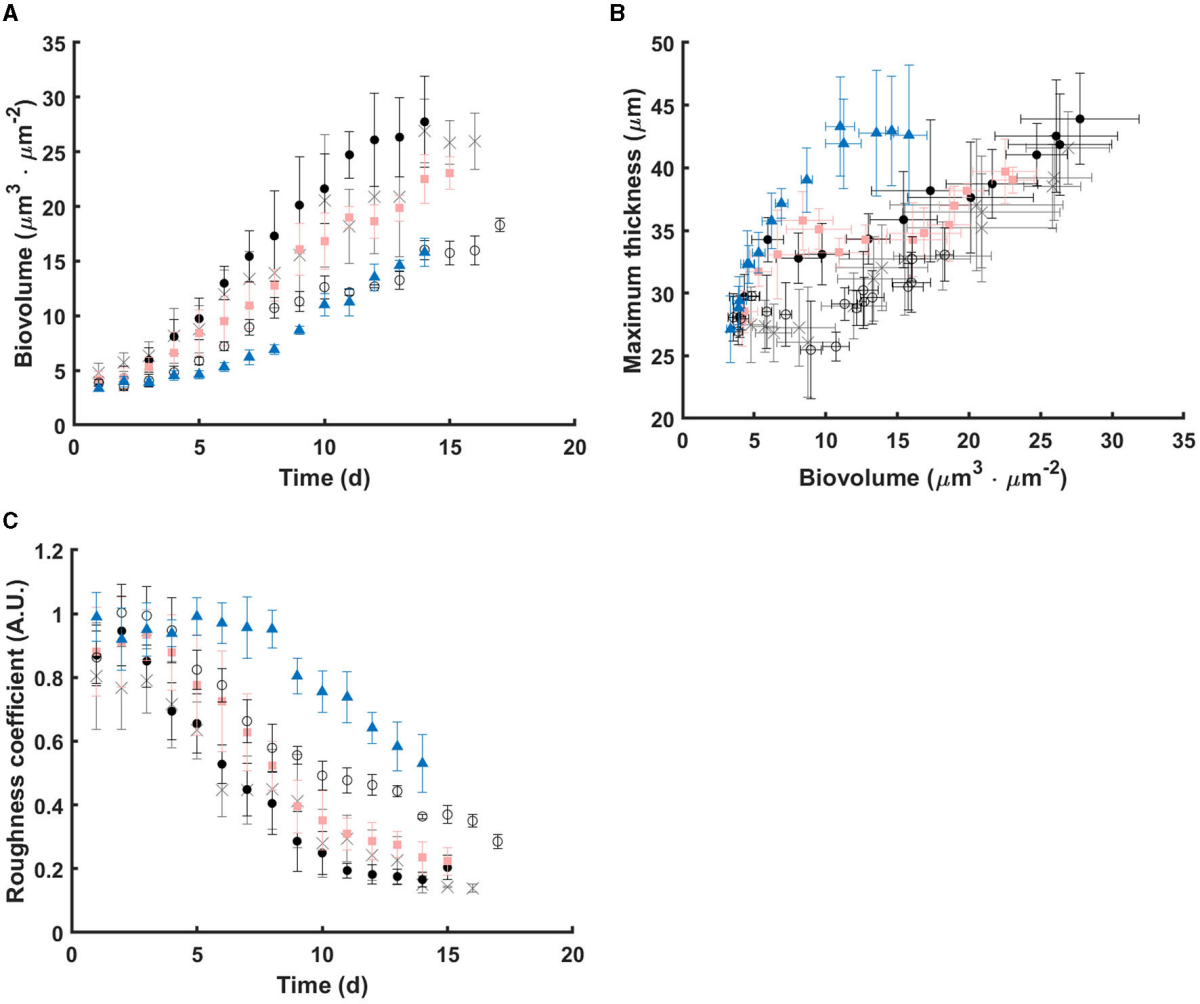
Dynamics of structural parameters under the tested light regimes. **(A)** Biovolume over time; **(B)** Relationships between Biovolume and Maximum thickness; **(C)** Roughness coefficient over time. Data points are the mean value of each parameter from replicates under continuous light (gray cross), 300-5s-0-10s (black dot), 300-30s-0-60s (pink square), 300-1min-0-2min (black circle), and 500-5s-0-20s (blue triangle), respectively. Errors bars represent the standard deviation of replicates.

Figure 1 displays representative images of 3D structure of *C. vulgaris* biofilms. Cell accumulation with time, due to cell division, is clearly visible. Interestingly, differences in structural dynamics can be detected among light conditions (Figure 2). Higher biovolume was observed for biofilms developed at 300-5s-0-10s from day 9 onwards, (p <0.05) compared with those exposed to equivalent continuous light intensity (Figure 2A). On the other hand, extended light/dark cycles in the minute scale impact growth negatively (*p* < 0.05), suggesting inhibition by light. In general, biofilms get smoother with time but significant structural pattern differences were observed among light regimes (see Figures 2B, C). Indeed, a sharp increase in the maximum thickness was observed in the initial stages of biofilm development for high peak PPFD at 500 μmol·m^−2^·s^−1^, even at low duty cycles, suggesting cell distribution in clusters (slope of maximum thickness over biovolume in 5 days: 2.78, adjusted *R*^2^: 0.95, see Supplementary material). By contrast, cells seem to distribute homogeneously on the support under continuous light (slope in 5 days: –0.25, adjusted *R*^2^: 0.39). Similar behavior has been described for *Botryococcus braunii* biofilms (Van Den Berg et al., 2019). Acclimation to low light (50 μmol·m^−2^·s^−1^) led to smaller and homogeneous colonies, whereas larger and heterogeneous colonies were found for middle (150 μmol·m^−2^·s^−1^) and high light (450 μmol·m^−2^·s^−1^) (Van Den Berg et al., 2019). Spatial cell distribution seems therefore light regime dependent.

### 3.2. Alternating light and darkness cycles affect biofilm growth and cell physiology

**Figure 3:**
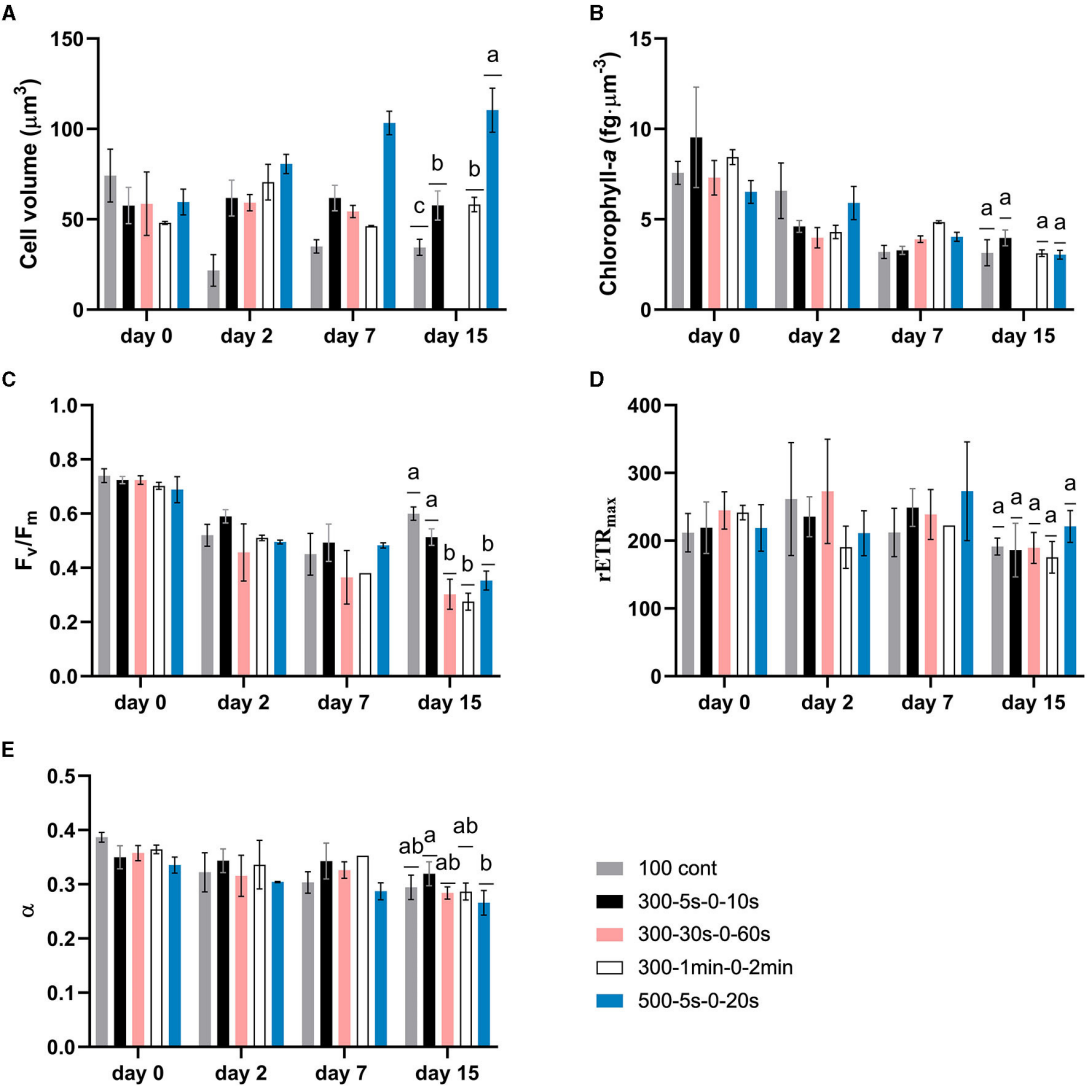
Dynamics of microalgae physiological parameters for biofilms under different light regimes. Data on “day 0” refer to photoacclimated suspended cells (inoculum culture). **(A)** average cell volume; **(B)** chlorophyll-a content normalized by per cell volume; **(C)** maximum quantum yield (*F*_*v*_/*F*_*m*_); **(D)** maximum relative electron transport rate; **(E)** initial slope of relative electron transport rate with respect to PPFD (α). Data are shown as mean ± standard deviation. Two-way ANOVA was carried out on each parameter, but statistics were only shown on day 15 with different letters representing the statistical differences among light regimes at the level of *P* < 0.05.

**Figure 4:**
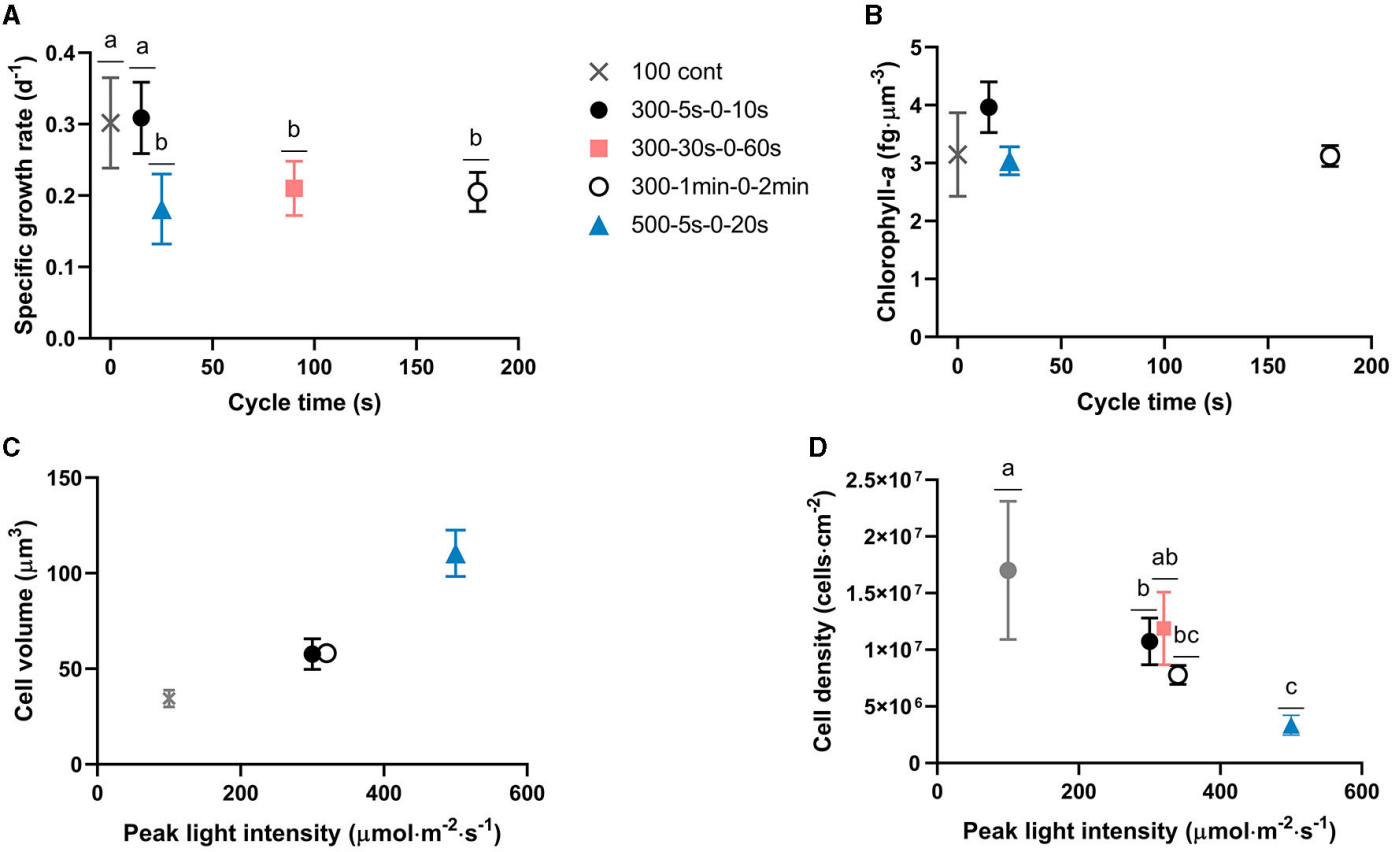
Cycle time and peak light intensity impacts on growth and physiology, respectively. **(A)** Biofilm specific growth rate and **(B)** Chlorophyll-a content with respect to cycle time; **(C)** Cell volume and **(D)** chlorophyll-a content per cell volume with respect to peak PPFD. Cell physiology parameters presented in this figure represent biofilm cell properties at day 15. Data points of the 3 light regimes with the same incident light of 300 μmol·m^−2^·s^−1^ shifted slightly to distinguish with each other in **(C, D)**. Bars with different letters represent the statistical differences among light regimes at the *P* < 0.05 level from the One-way ANOVA test. Data are shown as mean ± standard deviation.

In our study, full-light-integration is met up to cycle times of the order of tens of seconds (*p* > 0.05, Figure 4A). A decrease of the specific growth rate is though observed (*p* < 0.05) with extended cycle time certainly due to excess of light. This is in agreement with other studies demonstrating an improvement of biofilm photoefficiency and productivity with reduced cycle times (Toninelli et al., 2016). Regarding cell physiology, the evolution of several parameters (cell volume, chlorophyll-a content, *F*_*v*_/*F*_*m*_, α, and *rETR*_*max*_) is plotted with time for all tested regimes in Figure 3. First, a physiological shift from planktonic (day 0) to sessile state (day 2) is clearly observed, in particular, in terms of chlorophyll content (Figure 3B) and maximum quantum yield (Figure 3C). This behavior is in agreement with our previous works for biofilm cultivation in continuous light (Li et al., 2021). Second, a decreasing pattern in terms of chlorophyll-a content and *F*_*v*_/*F*_*m*_ were reported with time in biofilms (Figure 3B). In agreement with Combe et al. (2015) (cycle time from 0.2 s to 3 s, light fraction from 0.4 to 0.67, average PPFD of 400 μmol·m^−2^·s^−1^), cellular chlorophyll-a content photoacclimated to the same level as under equivalent continuous light, for all the tested light regimes (Figure 4B). It is also worth noting that light regime deeply impacts cell volume (Figure 3A). The interpretation of cell volume is complicated since it results from two concurrent mechanisms. The somatic growth is related to the necessity to accumulate the organic carbon produced by photosynthesis. Cell division leads to a reduction of cell size, and frequent division (high growth rate) resulting in smaller cells. In our study, growth rate is higher for continuous light and higher light frequency (at lower peak light), thus favoring smaller cell sizes. A deep decrease of cell volume is observed after 2 days for continuous light, followed by a slight increase over time. On the contrary, intermittent light at 300 μmol·m^−2^·s^−1^ peak light does not change much the cell volume. For the case of highest frequency, this suggests that lower cell size is compensated by a necessity to accumulate more photosynthetic products in the cell. In general, for these L/D cycles, the volume remains very close to that of the planktonic cells for all cycle times assessed. On the other hand, the cell behavior at 500 μmol·m^−2^·s^−1^ is totally different, with a marked increase of cell volume over time (two-fold increase in size of the cells). In this case, the high PPFD leads both to a lower growth rate, and most likely a higher energy flux in the cell during the light period, two factors leading to larger cells.

For all cycle times tested, the cell volume seems to be mainly driven by the peak PPFD (Figure 4C) and the necessity to accumulate on a short time scale storage compounds such as carbohydrates generated by the Calvin cycle. Interestingly, cell density is also peak light dependent (Figure 4D). This is consistent with other works demonstrating the influence of continuous light intensity on cell size (Winokur, 1948; Claustre and Gostan, 1987). A balance between light energy absorption and the overall utilization capacity of a cell must be maintained to optimize growth and protect the cell from excess of energy. In planktonic cultures of microalgae, the photo-acclimation state of the cells is not only reflected in different pigment contents and photosynthetic efficiency, but often also in changes of their macromolecular composition (Halsey and Jones, 2015). Storage pools such as carbohydrates and lipids typically serve as carbon and energy sinks during unbalanced growth due to high excitation pressure and/or nutrient limitation (Claustre and Gostan, 1987; Yilancioglu et al., 2014). An increase of intracellular sugars (Han et al., 2015; Schulze et al., 2017) and glycerol (Xu et al., 2016) has also been reported as a photoprotection mechanism in strong flashing light to avoid the generation or accumulation of harmful reactive oxygen species (ROS). Though changes in the chemical composition of biofilm cells are poorly studied, in particular in fluctuating light conditions, some works (Li et al., 2021) claim that mechanisms are similar to those involved in planktonic cultures. On the whole, there are two main mechanisms to manage the excess of light energy entering the cell during the short illumination period. Dissipating this energy through photosynthesis, while keeping the photosynthetic properties (chlorophyll content, see Figure 3B, and activity, see Figures 3D, E) for using the excess of light to produce storage compounds, consequently increasing in size. In addition, non-photochemical quenching mechanisms are known to be activated to earlier dissipate the excess excitation energy from the reaction center of PSII as already confirmed in *Chlorella* sp. biofilms (Wang et al., 2021). In our study, it is thus hypothesized that stressed cells exposed to high peak light intensity/low frequency are likely changing metabolism in order to use excess of light to produce storage compounds instead of dividing. However, the occurrence of non-photochemical quenching mechanisms is not ruled out. Parameters such as NPQ, carotenoids content, macromolecular (carbohydrates, lipids, proteins) composition should be therefore considered in future research to verify our hypothesis. The higher stress generated by unbalanced light regimes could also explain the formation of clusters, clearly observed for the 500 μmol·m^−2^·s^−1^ test. The cells at the surface are dramatically exposed to this high flux of energy, and in the natural diversity of the population, only the ones with less photosynthetic pigment survive. The top cells act as a protective shield to those lying underneath. They then allow cells to grow below, protecting them from this excess of light, naturally generating vertical structures. Such a photoprotection mechanism has been proposed in planktonic *Bracteacoccus aggregatus* cultures in response to UV-A stress (Chekanov et al., 2022). *B. aggregatus* aggregates with external bleached or intensely red-colored cells, due to carotenoid accumulation, and green internal cells, able to divide, were described in this work. Our observations are also in agreement with other of our studies describing the impact of light on the shape of immobilized cultures (Zhang and Perré, 2020). Additionally, to cope with high light stress, cell surface composition may also change consequently affecting cell-cell interactions and possible clustering, as previously described by Yuan et al. (2021).

### 3.3. Photoinhibition is mitigated in alternating light regimes

**Figure 5:**
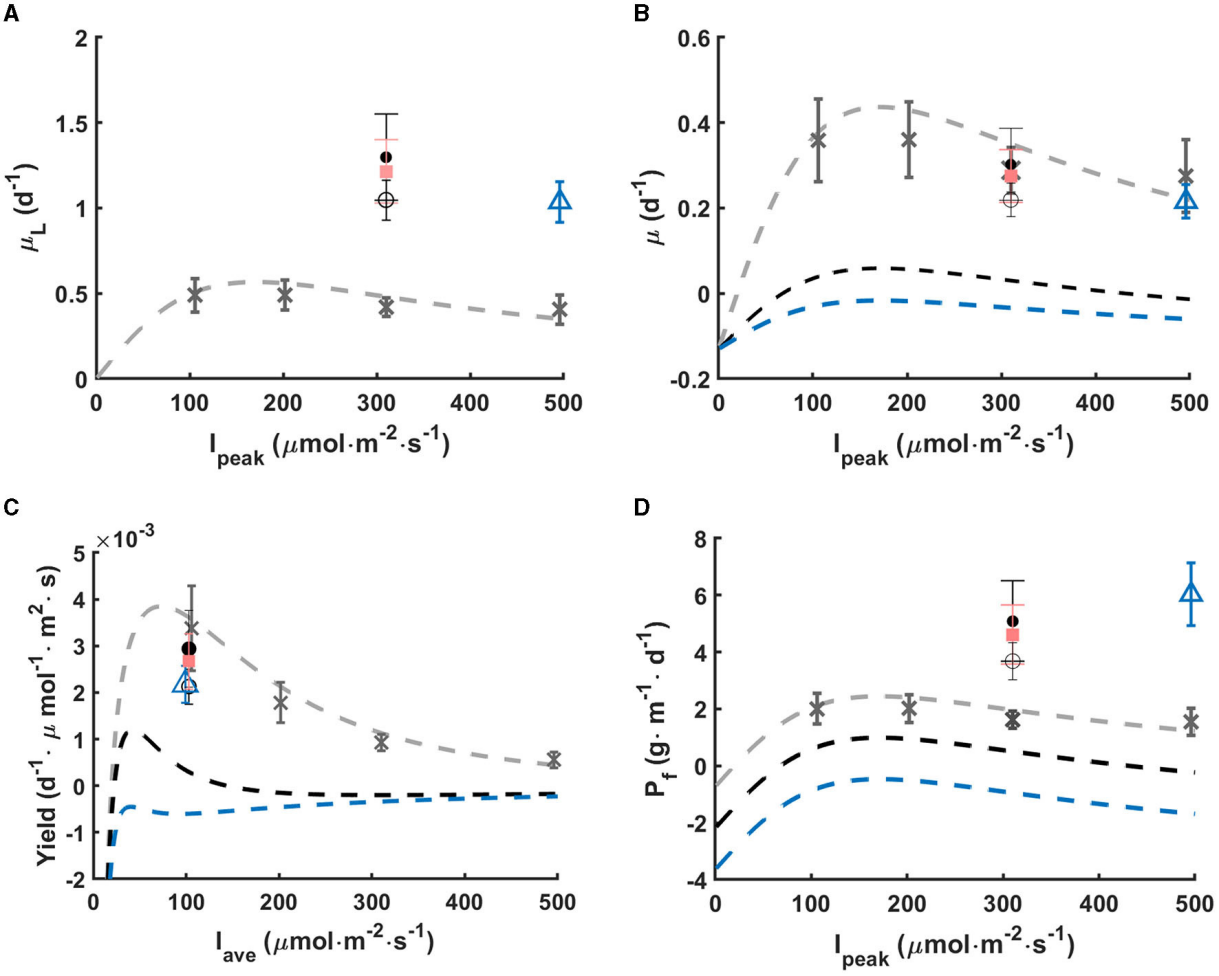
In rotating biofilm systems: **(A)** gross growth rate in light phase (μ_*L*_), **(B)** net growth rate (μ) with respect to peak light intensity, **(C)** growth yield with respect to average light intensity, and **(D)** net footprint productivity (*P*_*f*_) with respect to peak light intensity. Data points represent the mean values of μ_*L*_ and *P*_*f*_ calculated according to Sections 2.7, 2.9. Errors bars represent the standard deviation. Different light regimes are studied: continuous light (gray cross), 300-5s-0-10s (black dot), 300-30s-0-60s (pink square), 300-1min-0-2min (black circle), and 500-5s-0-20s (blue triangle). Dash lines represent the Haldane model fitting under continuous light (gray), and under intermittent light without inhibition mitigation with light fractions of 1/3 (black) and 1/5 (blue).

Intermittent light regimes tested in this work turn out to reduce photoinhibition induced by high light (300 and 500 μmol·m^−2^·s^−1^). This is shown in Figure 5A where computations of gross growth rate during the light phase μ_*L*_ are shown, assuming a constant respiration rate *R* during the light and dark phase. The net growth rates measured under various continuous light are also shown for comparison. It turns out that the μ_*L*_ recorded for the various light regimes is much larger than the one which would be expected for a large cycle time, *i.e.*, if growth rate during the light phase was the same as the one in continuous light with the same light intensity. The μ_*L*_ is more than twofold the one obtained for the same continuous light, which shows strong mitigation of the photoinhibition. This effect is even more marked when the net growth rate (μ) is plotted as a function of the peak light (see Figure 5B): the net growth rate is marginally affected by the periodic absence of light, and is not markedly different from the growth rate at constant peak light. Computing the apparent light yield (ratio of the net growth rate over the average light, see Figure 5C) it turns out that the yield in periodic light stays close to one observed at 100 μmol·m^−2^·s^−1^. Diluting light in time avoids the reduced yield due to photoinhibition. Cycle time controls the growth rate enhancement. With the same peak light of 300 μmol·m^−2^·s^−1^, the value of μ_*L*_ increases with shortened cycle time from 3 min to 15 s (*p* < 0.05), though the average light input is the same at 100 μmol·m^−2^·s^−1^.

In the work of Grenier et al. (2019), cycle times in the order of 30 min and 40 min led to a similar μ_*L*_ as for constant illumination, showing that photoinhibition was not mitigated. This case is also presented on Figure 5B to better understand how short cycles enhance photoefficiency. Here, with short cycle times, photoinhibition was significantly mitigated under all L/D cycles (*p* < 0.05).

There is another benefit in this approach. When considering a rotating system (Gross et al., 2013), with ε = 1/3, it means that the system contains 3 times more biomass than a system with ε = 1 which would be permanently exposed to full sunlight. Figure 5D shows that productivity is considerably enhanced for the same PPFD impinging the enlighten part of the biofilm. The cornerstone of this process is to cumulate the light mitigation effect, and the increase in total biomass per footprint unit.

The L/D cycles investigated in this work are representative of the cycles that could be obtained by rotating devices considering the shear stress (Gross et al., 2013) and energy consumption. Understanding and quantifying their impact on biofilm development is of paramount importance to help the operator to identify stressful parameters in advance, to spot optimal operating conditions and improve the reactor design. The best trade-off between productivity (high rotation speed to reduce the cycle time) and energy consumption (low rotation speed) can be more objectively determined. Our study confirms that short cycle times (in the order of tens of seconds) must be applied when aiming at maximizing cell productivity. This opens new routes for the production of high-value metabolites. Modeling has also to be promoted to integrate these findings into an operational tool for the design and operation of rotating biofilm systems.

## Data availability statement

The original contributions presented in the study are included in the article/Supplementary material, further inquiries can be directed to the corresponding author.

## Author contributions

YG, FL, and AF conceived the experiment. FL and AF contributed to the supervision of the experiments. YG, FL, AF, PP, and OB analyzed the results. YG wrote the first manuscript version which was updated by all authors. All authors contributed to the article and approved the submitted version.
