# Supplementary material for: The impact of light/dark regimes on structure and physiology of Chlorella vulgaris biofilms
Source: Front Microbiol. 2023 Oct 24;14:1250866. doi: 10.3389/fmicb.2023.1250866 (PMC10628651; doi:10.3389/fmicb.2023.1250866)
Supplement: Supplementary file 1 [file Data_Sheet_1.pdf]

Supplementary Material

SUPPLEMENTARY TABLES AND FIGURES

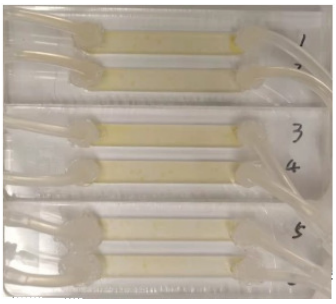

Figure S1. Three flow-cells placed parallel as one independent assay, with biofilms developing inside the channels.

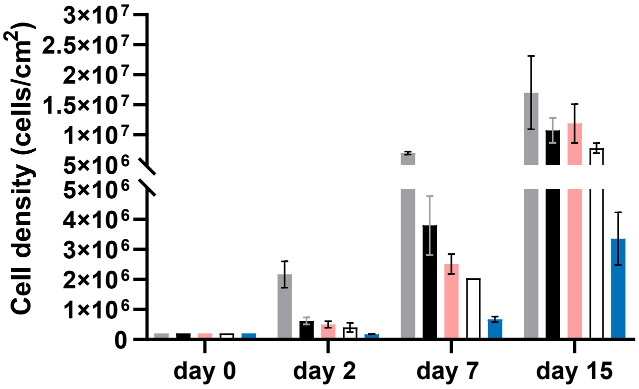

Figure S2. Cell density dynamics under five tested light regimes: 100 cont (gray), 300-5s-0-10s (black), 300-30s-0-60s (pink), 300-1min-0-2min (white), 500-5s-0-20s (blue). Data are shown in mean $\pm$ standard deviation.

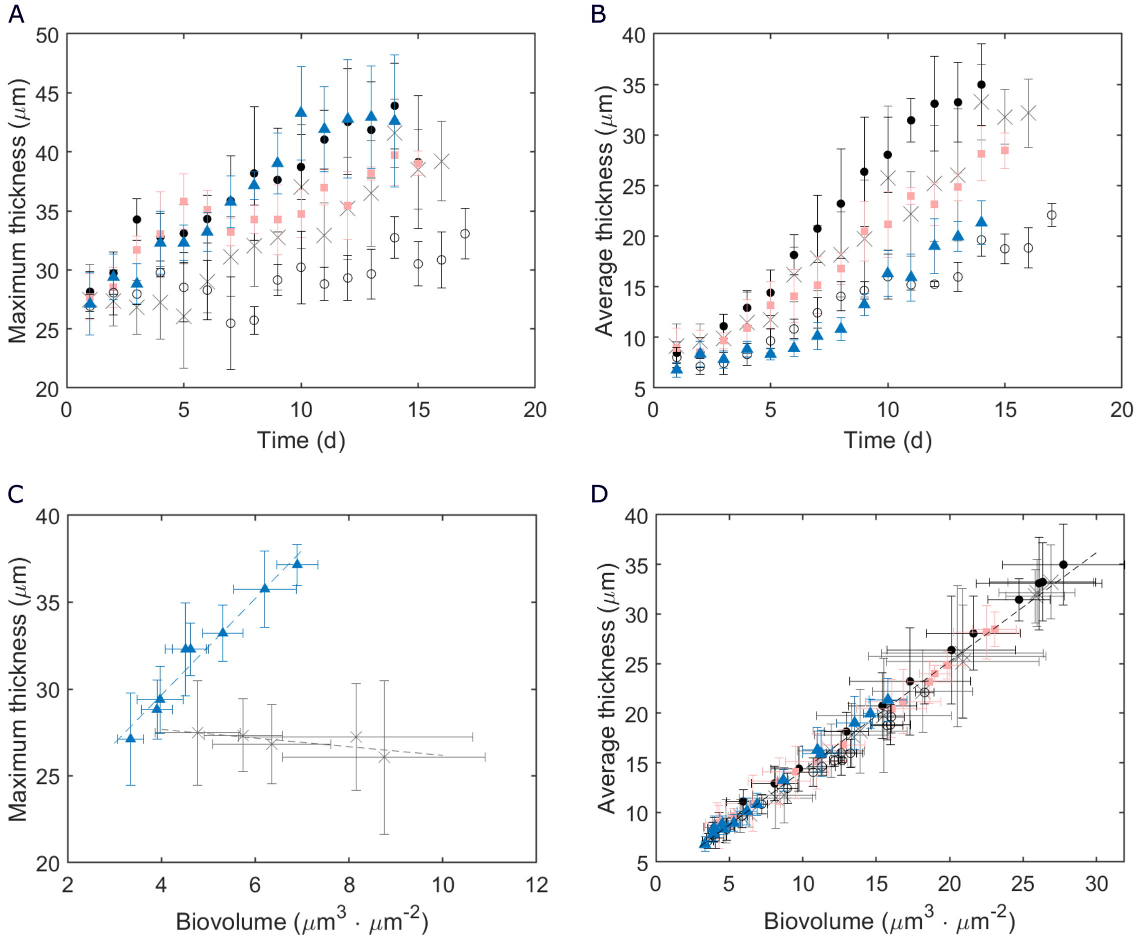

Figure S3. (A) Maximum thickness (μm) dynamics. (B) Average thickness (μm) dynamics. (C) Linear regression of maximum thickness over biovolume in light regimes of 100 cont (gray dash line: $y = -0.25x - 28.67$ with an adjusted R^2 of 0.39) and of 500-5s-0-20s (blue dash line: $y = 2.78x - 18.55$ with an adjusted R^2 of 0.95). (D) Linear regression of average thickness over biovolume in five tested light regimes (black dash line: $y = 1.098x + 3.25$ with an adjusted R^2 of 0.99). Different symbols represent the different light regimes: 100 cont (gray cross), 300-5s-0-10s (black dot), 300-30s-0-60s (pink square), 300-1min-0-2min (black circle), and 500-5s-0-20s (blue triangle). Data are shown in mean ± standard deviation.

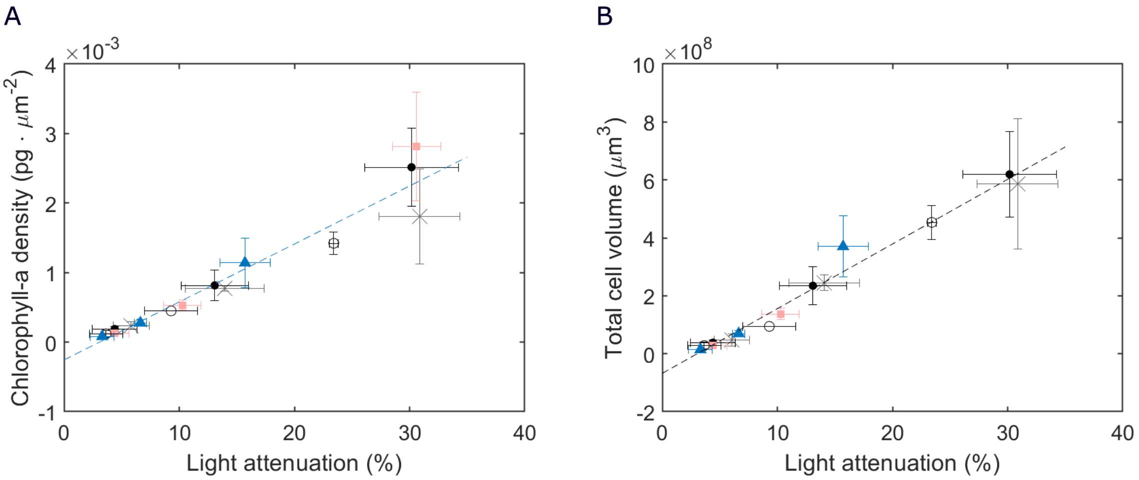

Figure S4. Chlorophyll-a density and the total cell volume were compared to the light attenuation for the culture at that time. Linear correlations were found suggesting that biofilm properties are stable over time. (A) Correlation between light attenuation at light spectra range from 400 nm to 700 nm and chlorophyll-a density (cellular chlorophyll-a content $(\mathrm{pg} \cdot \mathrm{cell}^{-1})$ $\times$ cell density from each time point) on the substratum. The correlation follows a linear relationship: $y = 8.34 \times 10^{-5}x - 2.57 \times 10^{-4}$ with an adjusted R^2 of 0.93. (B) Correlation between light attenuation and total cell volume (average cell volume $\times$ cell density from each time point). The correlation follows a linear relationship: $y = 2.23 \times 10^{7}x - 6.79 \times 10^{7}$ with an adjusted R^2 of 0.98. Different symbols represent data (mean $\pm$ standard deviation) from different light regimes: 100 cont (gray cross), 300-5s-0-10s (black dot), 300-30s-0-60s (pink square), 300-1min-0-2min (black circle), and 500-5s-0-20s (blue triangle).

Table S1. Parameters' definitions, values and units of Haldane model.

Parameters	Definition	Value	Unit
μ_{max}	maximum gross growth rate	$6.56 \cdot 10^{-6}$	s^{-1}
I_{opt}	optimal light intensity	171	$\mu\mathrm{mol} \cdot \mathrm{m}^{-2} \cdot \mathrm{s}^{-1}$
α	initial slope of the $\mu - I$ curve	$7.78 \cdot 10^{-8}$	$\mu\mathrm{mol}^{-1} \cdot \mathrm{m}^{2}$
R	respiration rate	$1.59 \cdot 10^{-2}$	s^{-1}

Table S2. Parameters' definitions, values and units for footprint productivity simulation.

Parameters	Definition	Value	Unit
h	biofilm thickness	$4 \cdot 10^{-5}$	m
S_f	footprint of biofilm reactor	1	m^2
ρ	biomass concentration	$1.40 \cdot 10^{5}$	$\mathrm{g} \cdot \mathrm{m}^{-3}$
